# Revealing Causes for False-Positive and False-Negative Calling of Gene Essentiality in Escherichia coli Using Transposon Insertion Sequencing

**DOI:** 10.1128/msystems.00896-22

**Published:** 2022-12-12

**Authors:** Donghui Choe, Uigi Kim, Soonkyu Hwang, Sang Woo Seo, Donghyuk Kim, Suhyung Cho, Bernhard Palsson, Byung-Kwan Cho

**Affiliations:** a Department of Bioengineering, University of California San Diego, La Jolla, California, USA; b Department of Biological Sciences and KI for the BioCentury, Korea Advanced Institute of Science and Technology, Daejeon, Republic of Korea; c School of Chemical and Biological Engineering, Seoul National University, Seoul, Republic of Korea; d School of Energy and Chemical Engineering, Ulsan National Institute of Science and Technology, Ulsan, Republic of Korea; e Department of Pediatrics, University of California San Diego, La Jolla, California, USA; Pontificia Universidad Catolica de Chile

**Keywords:** gene essentiality, subgenic-level essentiality, Tn-Seq, DNA-binding proteins, nucleoid-associated proteins

## Abstract

The massive sequencing of transposon insertion mutant libraries (Tn-Seq) represents a commonly used method to determine essential genes in bacteria. Using a hypersaturated transposon mutant library consisting of 400,096 unique Tn insertions, 523 genes were classified as essential in Escherichia coli K-12 MG1655. This provided a useful genome-wide gene essentiality landscape for rapidly identifying 233 of 301 essential genes previously validated by a knockout study. However, there was a discrepancy in essential gene sets determined by conventional gene deletion methods and Tn-Seq, although different Tn-Seq studies reported different extents of discrepancy. We have elucidated two causes of this discrepancy. First, 68 essential genes not detected by Tn-Seq contain nonessential subgenic domains that are tolerant to transposon insertion, which leads to the false assignment of an essential gene as a nonessential or dispensable gene. These genes exhibited a high level of transposon insertion in their subgenic nonessential domains. In contrast, 290 genes were additionally categorized as essential by Tn-Seq, although their knockout mutants were available. The comparative analysis of Tn-Seq and high-resolution footprinting of nucleoid-associated proteins (NAPs) revealed that a protein-DNA interaction hinders transposon insertion. We identified 213 false-positive genes caused by NAP-genome interactions. These two limitations have to be considered when addressing essential bacterial genes using Tn-Seq. Furthermore, a comparative analysis of high-resolution Tn-Seq with other data sets is required for a more accurate determination of essential genes in bacteria.

**IMPORTANCE** Transposon mutagenesis is an efficient way to explore gene essentiality of a bacterial genome. However, there was a discrepancy between the essential gene set determined by transposon mutagenesis and that determined using single-gene knockout strains. In this study, we generated a hypersaturated Escherichia coli transposon mutant library comprising approximately 400,000 different mutants. Determination of transposon insertion sites using next-generation sequencing provided a high-resolution essentiality landscape of the E. coli genome. We identified false negatives of essential gene discovery due to the permissive insertion of transposons in the C-terminal region. Comparisons between the transposon insertion landscape with binding profiles of DNA-binding proteins revealed interference of nucleoid-associated proteins to transposon insertion, generating false positives of essential gene discovery. Consideration of these findings is required to avoid the misinterpretation of transposon mutagenesis results.

## OBSERVATION

Determination of the essential set of genes encoded in a bacterial genome is an important part of understanding the genotype-phenotype relationship, engineering their metabolic capabilities, and creating synthetic bacterial genomes (1, 2). Several methods have been devised to discriminate between the essential and dispensable genes in a bacterium (3–6). Among those methods, transposon mutagenesis coupled with next-generation sequencing (Tn-Seq) identifies dispensable genes based on the generation of disruptive genomic insertions in a high-throughput manner (7–9). However, essential genes determined by transposon mutagenesis (10) differ from those determined by conventional knockout studies (4, 11, 12). Although some studies have reported a low rate of discrepancy (13), it varies from study to study (10), indicating there may be an inherent limitation to the method. The discrepancy originates from different sources, such as domain essentiality, sequence preference of transposon, and nucleoid-associated protein (NAP) interference (14–17). Here, we have revisited the relationship between the lack of transposon insertion and the interaction of NAPs with the genome for the accurate determination of essential genes in Escherichia coli K-12 MG1655 using a hypersaturated transposon mutant library.

**FIG 1:**
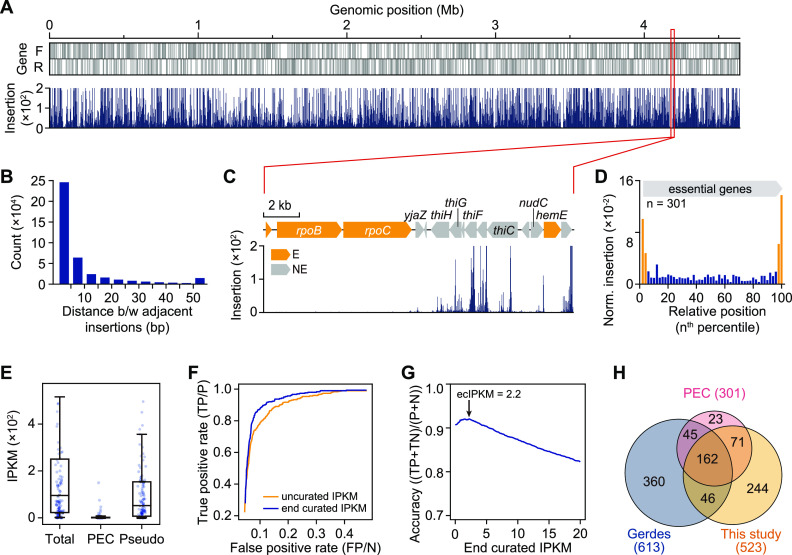
Genome-wide determination of gene essentiality using Tn-Seq. (A) Transposon insertion landscape of the E. coli MG1655 genome. Insertion profiles shown in this figure indicate normalized insertion (insertions per million insertions). (B) Histogram showing distances between two adjacent insertions. Over 90% of the insertion had an adjacent insertion within 25 bp. (C) Essential genes were not tolerant to transposon insertion due to disruptive properties of the transposon. E and NE indicate essential and nonessential genes, respectively, as reported in the PEC database. (D) Transposon insertion profile of PEC essential genes. (E) IPKM distribution of the total 4,498 genes, PEC essential genes, and pseudogenes. (F) A receiver operating characteristic (ROC) curve of IPKM when discovering essential genes. (G) Accuracy of essential gene discovery with different ecIPKM cutoffs. (H) Comparison between PEC essential genes and essential genes determined by previous Tn-Seq (Gerdes) and by this study, showing considerable discrepancies between experiments. Six phantom genes (b0322, b1228, b2612, b2651, b3112, and b4229) were excluded from the analysis.

We constructed 1 million transposon insertion mutants of E. coli K-12 MG1655 capable of growing on solid LB medium (see Text S1 in the supplemental material). Unique transposon insertion sites (TISs) across its genome were determined by using massively parallel sequencing. From 2.4 × 10^6^ mapped reads, a high-resolution transposon insertion landscape consisting of 400,096 unique TISs was obtained (Fig. 1A). TISs were distributed evenly across the genome, so that 89.6% had an adjacent TIS within 10 bp (Fig. 1B). Furthermore, GC content near TISs was 55.0%, which indicated that insertion bias based on GC content was negligible, considering that the GC content of the genome is 50.8%. Also, genomic regions with high or low GC content (>65% or <15%) showed no lack of transposon insertions, which are known to be depleted during amplification (Fig. S1 and Text S1) (18). As expected, we observed that essential genes, such as RNA polymerase subunits (*rpoBC*), had low transposon insertion frequencies (TIFs), whereas the nonessential genes had higher TIFs (Fig. 1C) (10, 19, 20).

10.1128/msystems.00896-22.1 TEXT S1 Supplemental materials, methods, and discussion. Download Text S1, DOCX file, 0.1 MB. Copyright © 2022 Choe et al. 2022 Choe et al. https://creativecommons.org/licenses/by/4.0/ This content is distributed under the terms of the Creative Commons Attribution 4.0 International license.

10.1128/msystems.00896-22.2 FIG S1 Assessment in GC bias of Tn*5* insertions or PCR amplification of the Tn-Seq library. (A) GC contents near transposon insertion positions. Genomic regions spanning 20 bp (nucleotide [nt] −10 to +10 from insertion), 40 bp, and 100 bp (window size) were tested. (B) IPKM distribution of genomic regions with high GC content (>65%). Box limits, whiskers, and center lines indicate first and third quartiles, 10th and 90th percentiles, and median of a distribution, respectively. Download FIG S1, JPG file, 1.0 MB. Copyright © 2022 Choe et al. 2022 Choe et al. https://creativecommons.org/licenses/by/4.0/ This content is distributed under the terms of the Creative Commons Attribution 4.0 International license.

To measure the TIF of each gene, we defined an insertion per kilobase per million insertions (IPKM) value, which is a modification of the widely used reads per kilobase per million mapped reads (RPKM) metric (Fig. S2A and B, Text S1) (21). The TISs that mapped onto either end of genes (4% at each end) were excluded from TIF calculation, since essential genes are tolerant to such insertions (Fig. 1D and Fig. S2C). To test the robustness of the end-curated IPKM (ecIPKM) to estimate gene essentiality (Table S1), a known set of essential genes in E. coli according to the Profiling of Escherichia coli Chromosome (PEC) database (11) and pseudogenes were examined (Fig. 1E). The ecIPKM of all genes ranged from 0 to 7,208.7 (median of 95.2), whereas PEC genes had very low ecIPKM values (median of 0.858). In contrast, pseudogenes exhibited high ecIPKM values (median of 52.1), in agreement with the fact that these genes are regarded as nonfunctional.

10.1128/msystems.00896-22.3 FIG S2 Examination of the ecIPKM metric. (A and B) Comparison between the insertion index metric and ecIPKM metric. The two metrics had a very high correlation (Pearson’s *R* of 0.717, Spearman’s *R* of 0.945) (A), and the insertion index and ecIPKM values of PEC essential genes had an even higher correlation (Pearson’s *R* of 0.947) (B). (C) End-curation accounted for transposons inserted at both ends of an essential gene. (D) IPKMs of PEC essential genes were markedly decreased after gene end-curation. μ indicates the average. (E) Distribution of the IPKMs of total genes was not changed before (raw) and after (curated) exclusion of gene ends. μ indicates average. (F) Number of essential and nonessential genes determined by given ecIPKM cutoff and categorized as true or false based on the PEC dataset. (G) Accuracy and coverage of PEC essential gene discovery with different ecIPKM cutoffs. Download FIG S2, JPG file, 2.4 MB. Copyright © 2022 Choe et al. 2022 Choe et al. https://creativecommons.org/licenses/by/4.0/ This content is distributed under the terms of the Creative Commons Attribution 4.0 International license.

10.1128/msystems.00896-22.7 TABLE S1 Gene essentiality determined by Tn-Seq (PEC, gene essentiality categorization according to the Profiling of E. coli Chromosome [PEC] database; NE, nonessential gene; E, essential gene; Gerdes, gene essentiality determined by previous Tn-Seq [Gerdes et al., 2003]; IPKM, insertion per kilobase per million mapped reads; ec, calculation after end-curation). Download Table S1, XLSX file, 0.7 MB. Copyright © 2022 Choe et al. 2022 Choe et al. https://creativecommons.org/licenses/by/4.0/ This content is distributed under the terms of the Creative Commons Attribution 4.0 International license.

To define a threshold that determined essential genes, the accuracies of ecIPKM cutoffs discovering the PEC essential genes were examined. The end curation improved the accuracy of essential gene discovery (Fig. 1F) without affecting overall IPKM distribution (Fig. S2D and E). We defined ecIPKM of 2.2 as a cutoff where accuracy was maximized (Text S1, Fig. 1G, and Fig. S2F and G). Using this criterion, 523 genes were determined as essential, of which 233 were PEC essential genes (Fig. 1H and Table S1). Compared with a previous transposon mapping with lower mutant library density (10) and Tn-Seq (13), which reported 68.8% and 88.7% coverage of PEC genes with false discoveries (*n* = 406 and 92), respectively, we identified similar coverage (77.4%) and false discoveries (*n* = 290). Essential gene sets discovered by different approaches seemed to vary due to many factors, such as experimental procedures, conditional essentiality, and statistics of analysis (Text S1 and Fig. S3).

10.1128/msystems.00896-22.4 FIG S3 Comparison between essential genes determined by previous Tn-Seq (Goodall et al.) and by this study. Download FIG S3, JPG file, 1.1 MB. Copyright © 2022 Choe et al. 2022 Choe et al. https://creativecommons.org/licenses/by/4.0/ This content is distributed under the terms of the Creative Commons Attribution 4.0 International license.

### Tn-Seq failed to detect 68 essential genes.

Tn-Seq with the ecIPKM metric failed to detect 68 PEC essential genes (Fig. 1H). These false negatives have been investigated previously (13). The comparison revealed that 22 genes were also falsely classified. This was caused by tolerated insertions on subgenic elements of essential genes, a polar effect, and misclassification in the PEC data set, among other things. Manual inspection of the false negatives unique to this study revealed that the false classifications were due to the same causes (Text S1 and Table S2). Most of the false negatives were caused by insertions on nonessential domains of essential genes, as recapitulated in a complementation experiment (Fig. S4).

10.1128/msystems.00896-22.5 FIG S4 Examples of essential genes with transposon insertions that were tolerated due to domain essentiality. (A) C-terminal sulfatase domain of an essential gene, *yejM*, related to cardiolipin transport, is dispensable according to the Tn-Seq profile, while the transmembrane domain is essential. The position of premature translational termination mutation is marked as a red triangle. (B) Methionine-tRNA synthetase encoded by *metG* is composed of two protein domains. Beside the two, the tRNA-binding domain (tRNA_bind) is dispensable. For a complementation experiment, three different truncated forms of the gene (*tmetG*1 to -3) were constructed. (C) Genomic copy of *metG* could be disrupted in a strain carrying native *metG* and *tmetG*3 in plasmid. Two remaining truncated *metG* genes could not rescue the disruption. Download FIG S4, JPG file, 2.7 MB. Copyright © 2022 Choe et al. 2022 Choe et al. https://creativecommons.org/licenses/by/4.0/ This content is distributed under the terms of the Creative Commons Attribution 4.0 International license.

10.1128/msystems.00896-22.8 TABLE S2 68 PEC essential genes which were determined to be nonessential in this study (TI, tolerated insertions [caused by, for example, domain essentiality, transposon-free region]; NS, not strictly essential; M, miscellaneous cause; F, false classification in previous dataset). Download Table S2, XLSX file, 0.02 MB. Copyright © 2022 Choe et al. 2022 Choe et al. https://creativecommons.org/licenses/by/4.0/ This content is distributed under the terms of the Creative Commons Attribution 4.0 International license.

### DNA-binding proteins interfered with transposon insertion.

**FIG 2:**
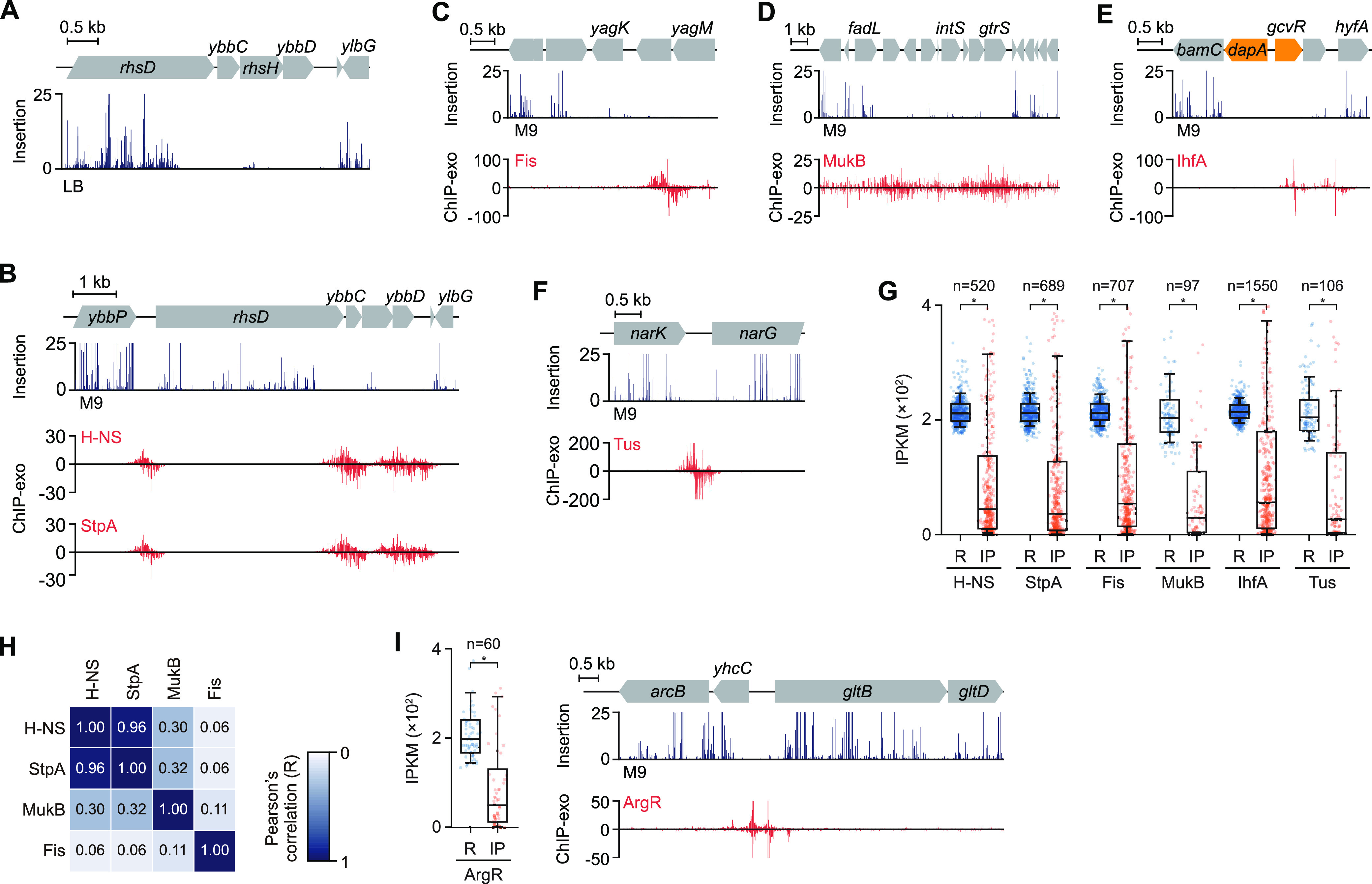
Interference of DNA-binding protein with transposon insertion revealed by ChIP-Exo. (A) Transposon insertion was not observed in the genomic region containing three nonessential genes, *ybbC*, *rhsH*, and *ybbD*. The profile shows Tn-Seq in LB medium. (B) Profiles show transposon insertion and binding of H-NS and StpA for three nonessential genes, *ybbC*, *rhsH*, and *ybbD*. H-NS and StpA binding on the three genes overlapped with the transposon-protected region. (C to F) Transposon insertion and ChIP-Exo profiles of Fis (C), MukB (D), IhfA (E), and Tus (F). (G) Box plot showing IPKM distribution of NAP-binding regions. Dots indicate IPKM of each region. As a control, the same-sized random genomic regions were tested. One of the 10,000 bootstrapped set is shown. R, random genomic regions; IP, NAP-binding regions determined by ChIP-Exo. Statistical significance was assessed by comparing the bootstrapped distribution of mean IPKM of 10,000 random sets. *, *P* < 0.05 (Welch’s *t* test). (H) Heatmap showing pairwise comparisons between H-NS, StpA, MukB, and Fis profiles. Pearson’s correlation coefficient (*R*) was calculated from ChIP-Exo intensity across the genome. Bindings of H-NS and StpA correlated with a coefficient of 0.955. MukB showed a relatively high correlation (>0.3) with H-NS and StpA, compared to Fis. (I) Box plot showing IPKM distribution of ArgR-binding regions; profiles show transposon insertion and ArgR binding near *gltB*. See the legend for panel G for details.

Next, we analyzed 290 false essential genes determined in the Tn-Seq library constructed from LB medium whose deletion mutants have been reported previously (Fig. 1H) (4, 11, 12). For example, a genomic region containing nonessential genes was protected from transposon insertion (Fig. 2A). We hypothesized that DNA-binding proteins (DBPs) can interfere with transposon insertion, as shown previously in different bacteria (16, 17). Specifically, we focused on NAP-DNA interactions, which were maintained similarly throughout the growth phase (22). Genome-wide binding regions of six NAPs in E. coli grown in M9 glucose medium were obtained by chromatin immunoprecipitation with exonuclease digestion (ChIP-Exo) (23). A total of 3,669 NAP-binding regions were detected (*q* < 0.05, Model-based Analysis of ChIP-Seq (MACS2) software) (Table S3) and compared with Tn-Seq data obtained from the same medium (Table S1). The NAP-binding regions perfectly overlapped with the protected regions from transposon insertion (Fig. 2B to F). Statistical analysis indicated the ecIPKM values of the NAP-binding regions were markedly lower (*P* < 0.05, Welch’s *t* test) than those for the random genomic regions (Fig. 2G). This was not due to specific bindings of NAPs to essential genes, because H-NS and StpA bound only 11 and 13 PEC genes, respectively, whereas the randomly sampled genomic regions overlapped 54.7 and 67.3 PEC genes on average, respectively. Overall, most of the false positives (238/290; 82.1%) contained NAP-binding regions covering more than 80% of the genic region, although the two data sets were collected from cells grown in different media. If we assumed the NAP binding was independent of medium, this would indicate that the interference is a major cause of false-positive discovery. The remaining 52 false positives were likely a result of DBPs that were not examined in this study that interfered with transposon insertion.

10.1128/msystems.00896-22.9 TABLE S3 Peaks detected by ChIP-Exo. Download Table S3, XLSX file, 0.1 MB. Copyright © 2022 Choe et al. 2022 Choe et al. https://creativecommons.org/licenses/by/4.0/ This content is distributed under the terms of the Creative Commons Attribution 4.0 International license.

Although we observed a few positions that were deprotected for transposon insertion in a *Δhns ΔstpA* double mutant (Fig. S5A and B and Table S4), transposon insertion of the NAP-binding regions was not fully relieved (Fig. S5C and D and Text S1), unlike the previous report on different bacteria (16). Even when the two NAPs were deleted simultaneously, other NAPs may have complemented the function of the two. MukB could be a candidate, as its binding profile is comparable to those of H-NS and StpA (*R* > 0.3) (Fig. 2H and Fig. S5E). In addition to the NAPs, the DNA-structuring transcriptional regulator ArgR (24) showed the same effect (Fig. 2I), which indicated that transcription factors also participated in this interference. We concluded that the interaction between DBPs and the bacterial genome interferes with transposon insertion, partially explaining the inconsistency between Tn-Seq and the knockout study when assessing gene essentiality. Overall, reevaluation of Tn-Seq results revealed that a consideration of interference of transposon insertion by DBPs is required to avoid misinterpretation of results. Unfortunately, there is no E. coli strain that lacks all the NAPs, nor is there a way of preventing DNA-NAP interactions to rapidly screen false essential genes. Thus, a high-density Tn-Seq experiment and careful evaluation of the results are necessary. Our study not only revealed the complexity of transposon insertion that leads to the identification of false essential genes but also has provided a high-resolution gene essentiality landscape of the E. coli genome.

10.1128/msystems.00896-22.6 FIG S5 Transposon insertion on NAPs and ArgR-binding regions in wild-type E. coli and deletion strains. (A) Transposons were inserted on H-NS and StpA binding regions in a double-knockout (DKO) strain, while it was protected in the wild-type strain. (B) Transposons were inserted on ArgR-binding region only in strain lacking ArgR. DKO, E. coli MG1655 *Δhns ΔstpA.* (C and D) IPKM distributions of H-NS binding regions in wild type or the DKO strain. Dots indicate IPKMs of each region. Diamonds represent means of distribution. As a control, 10,000 bootstrapped set of the same-sized random genomic regions were tested. R, random genomic regions; IP, NAP-binding regions determined by ChIP-Exo. Statistical significance was assessed by comparing the bootstrapped distribution of the mean IPKMs of 10,000 random sets. * *P =* 1.58 × 10^−10^; ns, *P =* 0.787 (Welch’s *t* test). (E) Correlation of binding profile of H-NS, StpA, and MukB. Download FIG S5, JPG file, 2.0 MB. Copyright © 2022 Choe et al. 2022 Choe et al. https://creativecommons.org/licenses/by/4.0/ This content is distributed under the terms of the Creative Commons Attribution 4.0 International license.

10.1128/msystems.00896-22.10 TABLE S4 Primers used in this study. Download Table S4, XLSX file, 0.04 MB. Copyright © 2022 Choe et al. 2022 Choe et al. https://creativecommons.org/licenses/by/4.0/ This content is distributed under the terms of the Creative Commons Attribution 4.0 International license.

### Data availability.

The sequencing data have been deposited in the European Nucleotide Archive (accession number PRJEB22130).
