# Supplementary material for: Revealing Causes for False-Positive and False-Negative Calling of Gene Essentiality in Escherichia coli Using Transposon Insertion Sequencing
Source: mSystems. 2022 Dec 12;8(1):e00896-22. doi: 10.1128/msystems.00896-22 (PMC9948719; doi:10.1128/msystems.00896-22)
Supplement: TEXT S1 [file msystems.00896-22-s0001.docx]

# Text S1

# Supplemental materials and methods

## Bacterial strains and culture conditions

*E. coli* K-12 MG1655 was used in this study, unless otherwise described. The cells were grown aerobically at 37 ºC on Luria-Bertani or M9 minimal medium (56.4 g/L M9 minimal salt, 2 mM MgSO_4_, 0.1 mM CaCl_2_, and 2 g/L glucose). Arginine was supplemented to a final concentration of 50 μM in M9 minimal medium, when appropriate.

## Complementation experiment of sub-genic essentiality of *metG*

Four different *metG* constructs were amplified from the genomic DNA of *E. coli* K-12 MG1655 using metG_F and one of the metG_R# primers, followed by cloning into pTak15K plasmid. The genomic copy of *metG* was deleted by chloramphenicol-resistant DNA cassette (amplified from pMloxC with the KO_metG_F and KO_metG_R primers) in a cell carrying pKD46 (1). Primer sequences are summarized in **Table S4**.

## Construction of knock-out strains

The knock-out strains were constructed by lambda recombination (1). The DKO strain (*E. coli* K-12 MG1655 *ΔhnsΔstpA*) was constructed by two sequential lambda recombinations (1). Briefly, the genomic copy of *hns* was substituted by a kanamycin-resistant DNA cassette (amplified from pKD13 using the KO_hns_F and KO_hns_R primers) in MG1655 carrying pKD46 plasmid (1). Then pCP20 (1), producing FLP recombinase, was introduced into the *hns::kan* strain to remove kanamycin-resistant cassette. The removal of the kanamycin cassette flanked by FLP recognition sites (FRT) was confirmed by kanamycin sensitivity and PCR genotyping. Then, pKD46 and pCP20 were cured from a successful deletion mutant by overnight incubation at 42 ºC. The genomic copy of *stpA* was removed by the repetition of the above procedure. Primer sequences are summarized in **Table S4**.

## Tn5-mutagenesis and genomic DNA isolation

The Tn5-transposon (Epicentre, EZ-Tn5™ <KAN-2> Tnp Transposome™ Kit) was transformed into *E. coli* K-12 MG1655 or DKO strain by electroporation and the transformed cells were selected on LB or M9 glucose agar plates containing kanamycin (50 μg/mL). Approximately 1 × 10^6^ transposon mutants (1 × 10^5^ for the DKO strain) were obtained on solid agar plate and collected into deep 96-well plates filled with 1.2 mL LB or M9 glucose medium (500 colonies per well). Aliquots of plates were stored at -80 °C after addition of 1.2 mL of sterile 50% glycerol solution. Genomic DNA was isolated from each well using Wizard Genomic DNA Purification Kit (Promega).

## Tn-Seq

The transposon-genome junction was amplified from extracted genomic DNA by a three-step PCR using HotStarTaq Master Mix (QIAGEN). All primers were added to give a final concentration of 0.5 μM. The first PCR was a semi-random PCR using Tn-Seq_F1 and one of the four Random_R# primers (**Table S4**). To avoid PCR bias, 100 ng of genomic DNA was used as a template. Four individual random reactions were separately amplified and combined prior to NGS. The Random_R# primers contained a 5 bp sequence motif derived from the MG1655 genome that represents every 1 kb, on average, and 8 bp degenerate N bases. HotStarTaq Master Mix (QIAGEN) was used for all PCR reactions. The first round of PCR was performed with the following conditions: (1) 95 ºC for 5 min, (2) 94 ºC for 20 s, (3) 63 ºC for 45 s, (4) 72 ºC for 3 min, (5) repeat of steps 2–4 for 9 cycles, (6) 94 ºC for 20 s, (7) 63 ºC for 45 s, (8) 72 ºC for 3 min, (9) 94 ºC for 20 s, (10) 63 ºC for 45 s, (11) 72 ºC for 3 min, (12) 94 ºC for 20 s, (13) 44 ºC for 1 min, (14) 72 ºC for 3 min, (15) repeat of steps 6–14 for 12 cycles, (16) 72 ºC for 7 min. One microliter of each first PCR product was diluted to 1:100 with nuclease-free water and used as template for the second PCR reaction. The second round of PCR was conducted with the Tn-Seq_F2 and Tn-Seq_R2 primers. The following program was used: (1) 95 ºC for 4 min, (2) 94 ºC for 20 s, (3) 63 ºC for 45 s, (4) 72 ºC for 5 min, (5) 94 ºC for 20 s, (6) 63 ºC for 45 s, (7) 72 ºC for 5 min, (8) 94 ºC for 20 s, (9) 53 ºC for 45 s, (10) 72 ºC for 5 min, (11) repeat steps 2-10 for cycles, (12) 94 ºC for 20 s, (13) 63 ºC for 45 s, (14) 72 ºC for 5 min, (15) 94 ºC for 20 s, (16) 53 ºC for 45 s, (17) 72 ºC for 5 min, (18) go to step 12, eight times, (19) 72 ºC for 7 min. The product of the second PCR was diluted 1:100 with nuclease-free water and used as a template for the third PCR. The third PCR was performed using Tn-Seq_F3 and Tn-Seq_R2 primers and the same reaction conditions as the second PCR. The products amplified by the same Random_R# primer were collected and DNA fragments between 150 and 600 bp were isolated using Pippin Prep DNA Size Selection System (Sage Science) equipped with a 2% agarose gel cassette in accordance with the manufacturer’s instructions. Equal amounts of four purified DNA samples were collected, based on measurements conducted by NanoDrop 2000 Spectrophotometer (Thermo Scientific). The final library was analyzed by TapeStation 2200 (Agilent) equipped with High Sensitivity D1000 ScreenTape (Agilent) prior to Illumina sequencing. The library was sequenced by Illumina MiSeq Instrument with a 150 cycle single-end recipe. Primer sequences are summarized in **Table S4**.

## ChIP-Exo

To identify *in vivo* NAP-binding, DNA-bound proteins were isolated from formaldehyde cross-linked *E. coli* cells by chromatin immunoprecipitation (ChIP) with an antibody that specifically recognizes the myc tag (9E10, Santa Cruz Biotechnology). The DNA-protein and antibody complex was isolated with Dynabeads Pan Mouse IgG magnetic beads (Invitrogen) followed by stringent washings, as previously described (2). Isolated chromatin was subjected to on-bead enzymatic reactions (3) with the following modifications. Briefly, the sheared DNA was repaired by the NEBNext End Repair Module (New England Biolabs) followed by the addition of a single dA overhang and ligation of the first adaptor (5′-phosphorylated) using dA-Tailing Module (New England Biolabs) and NEBNext Quick Ligation Module (New England Biolabs), respectively. Nick repair was performed by PreCR Repair Mix (New England Biolabs). Lambda exonuclease- and RecJf exonuclease-treated chromatin was eluted from the beads and the protein-DNA cross-link was reversed by overnight incubation at 65 ºC. RNA- and protein-removed DNA samples were used to perform primer extension and second adaptor ligation with the following modifications. The DNA samples incubated for primer extension were treated with the dA-Tailing Module (New England Biolabs) and NEBNext Quick Ligation Module (New England Biolabs) for second adaptor ligation. The DNA sample purified by GeneRead Size Selection Kit (Qiagen) was enriched by polymerase chain reaction (PCR) using Phusion High-Fidelity DNA Polymerase (New England Biolabs). The amplified DNA samples were purified again by GeneRead Size Selection Kit (Qiagen) and quantified by Qubit dsDNA HS Assay Kit (Life Technologies). The quality of the DNA sample was checked by an Agilent High Sensitivity DNA Kit using an Agilent 2100 Bioanalyzer (Agilent) before sequencing by MiSeq (Illumina) in accordance with the manufacturer’s instructions. Each modified step was also performed in accordance with the manufacturer’s instructions.

## Sequencing data processing

The sequencing data was processed on CLC Genomic Workbench (CLC Bio). Raw reads were quality trimmed by Trim Sequence Tool with a quality limit of 0.05 and reads with more than two ambiguous nucleotides were discarded. For ChIP-Exo, quality trimmed reads were mapped to the *E. coli* K-12 MG1655 genome sequence (NC_000913.3) with a mismatch cost of 2, InDel cost of 3, and length/similarity fraction of 0.9. For Tn-Seq, reads with the transposon sequence at their 5′ position were collected and the 5′ transposon sequence was trimmed by the Trim Adaptor object (5′-CCTGCAGGCATGCAAGCTTCAGGGTTGAGATGTGTATAAGAGACAG, strand: plus). The trimmed reads were mapped to *E. coli* K-12 MG1655 genome sequence (NC_000913.3) with mismatch cost of 2, InDel cost of 3, and length/similarity fraction of 0.8. Non-specific matches mapped randomly. Some of the unmapped reads had a sequencing adaptor read, because of their short insert length (< 150 sequencing cycles). Thus, the 3′ sequencing adaptor was trimmed from unmapped reads by the Trim Adaptor object (5′-CAAGCAGAAGACGGCATACGAGATCGGTCTCGGCATTCCTGCTGAACCGCTCTTCCGATCT, strand: minus). The trimmed reads were mapped again on the genome sequence with the same mapping parameters. Two consecutive mappings were merged and exported as a .BAM file. The BAM file was decompiled into .SAM plain text using the Samtools (v0.1.18) view command in a Linux environment (4). The 5′ end of the read denote for the transposon insertion site and were extracted from the SAM file. The extracted transposon insertion sites were encoded as a .GFF file format for visualization and the IPKM calculation was performed using an in-house python script. The GFF file was visualized on SignalMap (v2.0.0.5, Roche NimbleGen). NAP binding peaks were detected using Model-based Analysis for ChIP-Seq (MACS2) (5) with following options: genome size, 4.6E+6; nomodel, on. Suspiciously short and weak peaks in comparison with the background were discarded by the manual inspection of profiles.

## IPKM calculation

IPKM was calculated from the following equation:

$$IPKM=\frac{number of insertions on a target locus}{locus length \left( kb \right) \times total insertions on the genome (million)}$$

Multiple reads fall onto the same position were counted multiple times.

## Statistical analysis

The IPKM of the DNA-binding protein binding region was calculated with the same IPKM calculation for genes, except for end-curation. The IPKM of open genome (free of DNA-binding protein) could not be defined because IPKM calculates the insertion frequency of a defined genomic loci. Thus, a set of random genomic fragments composing the same number and length of the given NAP- or ArgR-binding regions were generated. To estimate IPKM distribution of random genomic region, the random set generation was repeated 10,000 times (bootstrapped). The bootstrap distribution of mean IPKM of random sets were compared to the IPKM distribution of NAP- or ArgR-binding regions using Welch’s *t* test.

# Examination of insertion and amplification biases of the dataset

Previously, it has been reported that high GC preference of Tn5 transposon (6). GC content near TIS detected in this study was approximately 55.0% regardless of window sizes tested (**Fig. S1A**) indicating the insertion bias is negligible considering the GC content of *E. coli* genome (50.8%). Saturated mutagenesis with enough library size seems to overcome the native bias of Tn5 transposon.

In addition, it has been known that GC-content of DNA generates considerable PCR bias when constructing high-throughput sequencing libraries. According to the previous literature, DNA with GC content higher than 65% or lower than 15% had lower amplification efficiency which made them depleted one-hundredth to a normal DNA after PCR amplification (7, 8). Thus, we examined IPKM distribution of genomic fragments with GC content higher or lower than 65% or 15%, respectively, with window sizes of 150, 300, 450, and 600 bp (which cover size of Tn-Seq library). First, there’s no genomic region with the given window size has GC content lower than 15% in *E. coli*. On the other hand, genomic regions containing high GC had tendency to have higher IPKM than genome-wide screen which is an opposite of what PCR bias supposed to be (**Fig. S1B**). Collectively, we concluded that there is no observable insertion or amplification bias in the dataset.

# IPKM metric and insertion index

As greatly reviewed previously (9), there are many different approaches in analyzing Tn-Seq dataset. Annotation independent methods, such as the sliding window (10) and statistical models (11, 12), are useful in detecting essentiality of sub-genic elements, such as protein domains. However, we did not analyze the dataset with the methods because of three reasons. First, the sliding window method divides genome into overlapping fragments of a specific size (10). It has never been examined how size of the sliding window impacts on the sensitivity and result of Tn-Seq analysis. We believe an optimal size of the sliding window may be different depending on many factors such as genome size and library density. Thus, we used an annotation-based measurement of insertion instead of using sliding window method with an arbitrary size (250-600 bp). Second, a probabilistic model, using Poisson model (12) or HMM (11) as an example, naturally generates false discoveries due to a multiple testing, especially as number of test increases. Thus, it necessitates correction for the multiple testing, therefore limits the statistical power of analysis (13). In addition, these models suffer from predicting essentiality of short genes. Lastly, considering there are only 68 of false negatives and 36 were related to domain essentiality, it is more reasonable to use simpler and straightforward method with handling rare exceptions, instead of using methods that potentially introduce statistical or modelling bias that are not perfectly represents a nature of Tn-Seq experiment.

Owing to the reasons listed above, we chose to measure annotation-based insertion frequency when analyzing the data. Previously, number of insertion sites in a gene, normalized by size of the gene, has been used, named insertion index, as a measure for transposon insertion frequency (14). In the metric, multiple reads mapped on an insertion site are counted as one. However, in IPKM metric, those reads are counted multiple times to account for the fitness effects of insertions which may provide more deciding power when detecting non-essential genes. To examine, we calculated insertion index of our dataset and compared it to ecIPKM metric (**Fig. S2A**). First of all, the main drawback of ecIPKM metric would be its higher sensitivity to duplicated reads during sequencing library amplification than insertion index. If there was a widespread read duplication during amplification or sequencing, there would be an overestimation of insertion frequency observed across entire insertion index range. However, we could not detect a consistent deviation of the dataset along with y-axis, indicating that there was no amplification issue during the library construction (**Fig. S2B**). In fact, distribution indicates that two metrics have very high correlation (Pearson’s R of 0.717; Spearman’s R of 0.945). Even for PEC essential genes, two metrics had even higher correlation (Pearson’s R of 0.947). The ecIPKM metric deviated to higher values at insertion index over 100. This phenomenon may be due to fitness effect of insertion mutants, whose inactivation is advantageous, outcompeting other mutants. Thus, we believe there would be minimal effect in determining essential gene, especially unlikely because the dataset had more linear correlation when genes had fewer insertions.

# Determination of ecIPKM cutoff to distinguish between essential and non-essential genes

To define a threshold that determines essential genes, we compared the PEC essential genes with essential genes determined by applying ecIPKM cutoffs (from 0.1 to 100 with 0.1 increment). Accuracy of the essential gene discovery maximized at ecIPKM cutoff 2.2 (**Fig. 1G**). However, accuracy of ecIPKM cutoff between from 1 to 2.2 remained almost identical level (**Fig. 1G**). Further investigation of the dataset indicated that accuracy of gene essentiality determination is considerably dependent on non-essential genes as they are most of the cases (more than 4000 non-essential genes). Thus, we examined four different calls, true positives/negatives and false positives/negatives based on PEC dataset. Number of true essential genes (true positives) and false non-essential genes (false negatives) rapidly increases and decreases, respectively, as the cutoff value increases from 0 to 2 (**Fig. S2F**). At ecIPKM cutoff higher than 2, the true calls reached a plateau and number of false positives kept increasing linearly. Thus, an optimal cutoff could not be much larger than ecIPKM of 2 as false positives increased much faster than the true calls. Thus, we defined ecIPKM of 2.2 as a cutoff for essential gene determination because the accuracy was maximized without discovering too much false positives and covers a large proportion of PEC essential genes (**Fig. S2G**). However, one important thing to note is that there will be no precise number of ecIPKM that perfectly determines gene essentiality. There will be a certain level of arbitrariness near the cutoff because it is hard to distinguish genes whose disruption induces extreme growth retardation or complete lethality.

# Discrepancies between essential genes in *E. coli* determined by multiple Tn-Seq

Tn-Seq experiment performed in the same *E. coli* strain (K-12, MG1655) or a closely related K-12 strain *E. coli* BW25113 had different composition of essential genes than our dataset (**Fig. 1H** and **Fig. S3**). This is not surprising because there could be multiple causes of the inconsistency such as different experimental procedures, statistical analyses, culture conditions, strains, and suppressor mutations accumulated during cell propagation. We compared essential gene set determine by Goodall *et al.* to that of this study (**Fig. S3**), since the individual genes of the other dataset is not extensively reconciled. One hundred genes were unique to the previous report. Of 100, 52 were already investigated in the previous report that there was conditional essentiality or domain essentiality of sub-genic elements that make Tn-Seq to inaccurately estimate gene essentiality. Thirteen of the remaining had ecIPKM lower than two-fold cutoff (4.4). These genes fall into highly arbitrary position where slight changes in statistics and cutoff would classify them as essential or non-essential. Thus, we believe that this is a discrepancy in sensitivity of analysis. There remained 35 genes, many of whose knock-out mutants were reported previously (examples including, but not limited to, *aceF, ybeY, sucAB, rnt*) (15-18). This discrepancy may be a strain-specific essentiality difference due to different gene composition, suppressor mutations, and gene duplication events. In depth comparative genomic approach is required for further characterization.

On the other hand, 265 genes were uniquely essential in the present study. Except for the 72 genes that are not present in the *E. coli* BW25113 strain, majority of them (n=158) overlapped with 6 NAPs binding sites, which are discussed in the later in this paper. It is unknown whether NAPs binding events are different between in these *E. coli* strains, essential genes detected in present study were sensitive to NAPs binding. Interestingly, it is reported that bacteriophage T4 early protein MotB dysregulates silencing effect of H-NS (19). Thus, it is noteworthy that there may be an unknown plasticity of NAPs binding in *E. coli* that make dataset of Goodall *et al.* less sensitive to NAPs-DNA interaction. Besides, we would also hypothesize that differences in strain, dosage of transposome complex used for transformation, experimental conditions/procedures, transposon DNA sequence, antibiotics marker could induce this discrepancy. Next, seven of the remaining 35 genes were classified as ambiguous by Goodall *et al*, because likelihood of the genes being essential or non-essential group is inconclusive. Interestingly, remaining 28 genes were prophages or poorly characterized genes. Considering that NAPs play role in silencing foreign DNA, these genes may be also protected by NAPs that are not examined in this study (20). Investigation of the discrepancy between two datasets is interesting to us, however we concluded that it should be further deeply investigated in more controlled experimental setting, specifically designed to account for strain-specific differences, which is beyond scope of this study.

# Manual inspection revealed reasons of misclassification of 68 false negatives of Tn-Seq

The 68 false negatives arose from various biological events that led misclassification of gene essentiality. We compared false negatives with the previous report that investigated discrepancies between Tn-Seq and PEC dataset (21). Of 68 false negatives, 22 genes were also falsely classified as non-essential in the previous Tn-Seq although those genes are classified as essential in the PEC dataset (**Table S2**). Nine genes had transposon insertion free region indicating that there are essential sub-genic regions (such as protein domain) while remaining regions are tolerant to transposon insertion. For example, an essential gene *yejM*, related to LPS homeostasis (22), had transposon insertion only at the 3′ domain (**Fig. S4A**), which agreed with the previous report (23). Five genes (*priA*, *efp*, *ftsE*, *rsgA,* and *coaE*) are classified as essential in the PEC dataset; however, their knockout mutants were reported elsewhere or there is an error in library construction. This indicates that they were misclassified by the PEC dataset, which made the discrepancy (24-28). Remaining 8 genes were caused by miscellaneous reasons including polar effect of transcriptional interference, conditional essentiality difference, occurrence of suppressor mutations, and short length of the gene (**Table S2**) (29).

Remaining 46 false negatives that are unique to our dataset were classified as non-essential with similar causes. Twenty-seven genes had sub-genic regions that are free of transposon insertion while the remaining intragenic regions are tolerant to insertions (**Table S2**). For example, methionine-tRNA ligase, encoded by *metG,* had transposon insertion only at C-terminal portion of the gene (**Fig S4B**). We constructed strains heterologously expressing truncated forms of MetG (**Fig. S4C**). Strains expressing a full-length *metG* or *metG* lacking putative tRNA binding domain (30) could survive when the genomic copy was deleted. However, the genomic *metG* could not be deleted in the strains carrying the *metG* with broken tRNA-synthetase domain (**Fig. S4C**). Other than the domain essentiality, three genes, *gpsA*, *folD*, and *ubiA*, had insertions likely because of conditional difference in essentiality as *folDK* and *ubiBD* had in the previous report (21). Remaining 16 genes had an ecIPKM value below the two-fold threshold cutoff (4.4). Disruption of these genes may cause very weak growth, which lead to their categorization as essential in the previous studies.

# IPKM of H-NS binding sites in strain lacking *hns* and *stpA*

As the NAPs interfere with transposon insertion, we examined that deletion of NAPs would reduce the interference as reported previously in *Vibrio cholerae* (31). Although we could find de-protection in some of the H-NS binding sites (**Fig. S5**), it was not fully relieved. We calculated IPKM distribution of H-NS binding sites from Tn-Seq result of *ΔhnsΔstpA* strain. Technically, it is not possible to calculate IPKM of H-NS binding sites in the knockout strain, because there is no binding site in the strain. Thus, it is noteworthy that it would be a IPKM distribution of H-NS shadow in the knockout strain. Mean IPKM of H-NS shadow in the knockout strain was not significantly different from that of randomly chosen set (**Fig. S5C**). However, the distribution looks different due to the binding sites with low IPKM values. Fully shown distribution highlights the extremely high variance of the distribution (**Fig. S5D**). This is partly because of the smaller size of *ΔhnsΔstpA* transposon mutant library (composed of only 10^5^ mutants). Thus, we concluded that the deletion of *hns* and *stpA* did not resulted in nucleoid deformation of the *hns and stpA* binding sites*.* One possible explanation is more complex interplay between different NAPs and complexity of nucleoid in *E. coli* than *V. cholerae*. E. coli possesses 16 NAPs (MraZ, StpA, Fis, YejK, IhfA, Ssb, HupAB, MukBEF, RdgC, H-NS, YbaB, CbpA, SlmA, and Dps) according to the current annotation and Gene Ontology classification (GO:0009295 - nucleoid), whereas *V. cholerae* had only three NAPs, namely H-NS, YejK, and YbaB/EbfC family protein, and few auxiliary proteins such as nucleoid occlusion factor SlmA, chromosome partition proteins MukBEF. According to the ChIP-Exo profiles, H-NS, StpA, and MukB bindings are highly correlated (**Fig. 2H** and **Fig. S5E**), even though the nucleotide-level resolution ChIP-Exo profiles were compared. Thus, NAPs other than H-NS and StpA may complement the double knockout. Unfortunately, MukB could not be deleted to address this issue since it is an essential protein for survival. Thus, further investigation into dynamics and cooperative behavior between NAPs is required.

# Text S1 References

1. Datsenko KA, Wanner BL. 2000. One-step inactivation of chromosomal genes in *Escherichia coli* K-12 using PCR products. Proc Natl Acad Sci U S A 97:6640-5.

2. Cho BK, Barrett CL, Knight EM, Park YS, Palsson BO. 2008. Genome-scale reconstruction of the Lrp regulatory network in *Escherichia coli*. Proc Natl Acad Sci U S A 105:19462-7.

3. Rhee HS, Pugh BF. 2011. Comprehensive genome-wide protein-DNA interactions detected at single-nucleotide resolution. Cell 147:1408-19.

4. Li H, Handsaker B, Wysoker A, Fennell T, Ruan J, Homer N, Marth G, Abecasis G, Durbin R, Genome Project Data Processing S. 2009. The Sequence Alignment/Map format and SAMtools. Bioinformatics 25:2078-9.

5. Zhang Y, Liu T, Meyer CA, Eeckhoute J, Johnson DS, Bernstein BE, Nusbaum C, Myers RM, Brown M, Li W, Liu XS. 2008. Model-based analysis of ChIP-Seq (MACS). Genome Biol 9:R137.

6. Green B, Bouchier C, Fairhead C, Craig NL, Cormack BP. 2012. Insertion site preference of Mu, Tn5, and Tn7 transposons. Mob DNA 3:3.

7. Aird D, Ross MG, Chen WS, Danielsson M, Fennell T, Russ C, Jaffe DB, Nusbaum C, Gnirke A. 2011. Analyzing and minimizing PCR amplification bias in Illumina sequencing libraries. Genome Biol 12:R18.

8. Dabney J, Meyer M. 2012. Length and GC-biases during sequencing library amplification: a comparison of various polymerase-buffer systems with ancient and modern DNA sequencing libraries. Biotechniques 52:87-94.

9. Chao MC, Abel S, Davis BM, Waldor MK. 2016. The design and analysis of transposon insertion sequencing experiments. Nat Rev Microbiol 14:119-28.

10. Zhang YJ, Ioerger TR, Huttenhower C, Long JE, Sassetti CM, Sacchettini JC, Rubin EJ. 2012. Global assessment of genomic regions required for growth in *Mycobacterium tuberculosis*. PLoS Pathog 8:e1002946.

11. DeJesus MA, Ioerger TR. 2013. A Hidden Markov Model for identifying essential and growth-defect regions in bacterial genomes from transposon insertion sequencing data. BMC Bioinformatics 14:303.

12. Gerdes SY, Scholle MD, Campbell JW, Balazsi G, Ravasz E, Daugherty MD, Somera AL, Kyrpides NC, Anderson I, Gelfand MS, Bhattacharya A, Kapatral V, D'Souza M, Baev MV, Grechkin Y, Mseeh F, Fonstein MY, Overbeek R, Barabasi AL, Oltvai ZN, Osterman AL. 2003. Experimental determination and system level analysis of essential genes in *Escherichia coli* MG1655. J Bacteriol 185:5673-84.

13. Grenov AI, Gerdes SY. 2008. Modeling competitive outgrowth of mutant populations: why do essentiality screens yield divergent results? Methods Mol Biol 416:361-7.

14. Langridge GC, Phan MD, Turner DJ, Perkins TT, Parts L, Haase J, Charles I, Maskell DJ, Peters SE, Dougan G, Wain J, Parkhill J, Turner AK. 2009. Simultaneous assay of every Salmonella Typhi gene using one million transposon mutants. Genome Res 19:2308-16.

15. Kurono N, Matsuda A, Etchuya R, Sobue R, Sasaki Y, Ito M, Ando T, Maeda S. 2012. Genome-wide screening of *Escherichia coli* genes involved in execution and promotion of cell-to-cell transfer of non-conjugative plasmids: *rodZ* (*yfgA*) is essential for plasmid acceptance in recipient cells. Biochem Biophys Res Commun 421:119-23.

16. Rasouly A, Schonbrun M, Shenhar Y, Ron EZ. 2009. YbeY, a heat shock protein involved in translation in *Escherichia coli*. J Bacteriol 191:2649-55.

17. Yu BJ, Sung BH, Lee JY, Son SH, Kim MS, Kim SC. 2006. *sucAB* and *sucCD* are mutually essential genes in *Escherichia coli*. FEMS Microbiol Lett 254:245-50.

18. Hsiao YY, Fang WH, Lee CC, Chen YP, Yuan HS. 2014. Structural insights into DNA repair by RNase T--an exonuclease processing 3' end of structured DNA in repair pathways. PLoS Biol 12:e1001803.

19. Son B, Patterson-West J, Arroyo-Mendoza M, Ramachandran R, Iben JR, Zhu J, Rao V, Dimitriadis EK, Hinton DM. 2021. A phage-encoded nucleoid associated protein compacts both host and phage DNA and derepresses H-NS silencing. Nucleic Acids Res 49:9229-9245.

20. Navarre WW, McClelland M, Libby SJ, Fang FC. 2007. Silencing of xenogeneic DNA by H-NS-facilitation of lateral gene transfer in bacteria by a defense system that recognizes foreign DNA. Genes Dev 21:1456-71.

21. Goodall ECA, Robinson A, Johnston IG, Jabbari S, Turner KA, Cunningham AF, Lund PA, Cole JA, Henderson IR. 2018. The Essential Genome of *Escherichia coli* K-12. mBio 9:e02096-17.

22. De Lay NR, Cronan JE. 2008. Genetic interaction between the *Escherichia coli* AcpT phosphopantetheinyl transferase and the YejM inner membrane protein. Genetics 178:1327-37.

23. Clairfeuille T, Buchholz KR, Li Q, Verschueren E, Liu P, Sangaraju D, Park S, Noland CL, Storek KM, Nickerson NN, Martin L, Dela Vega T, Miu A, Reeder J, Ruiz-Gonzalez M, Swem D, Han G, DePonte DP, Hunter MS, Gati C, Shahidi-Latham S, Xu M, Skelton N, Sellers BD, Skippington E, Sandoval W, Hanan EJ, Payandeh J, Rutherford ST. 2020. Structure of the essential inner membrane lipopolysaccharide-PbgA complex. Nature 584:479-483.

24. Balibar CJ, Iwanowicz D, Dean CR. 2013. Elongation factor P is dispensable in *Escherichia coli* and *Pseudomonas aeruginosa*. Curr Microbiol 67:293-9.

25. de Leeuw E, Graham B, Phillips GJ, ten Hagen-Jongman CM, Oudega B, Luirink J. 1999. Molecular characterization of *Escherichia coli* FtsE and FtsX. Mol Microbiol 31:983-93.

26. Hase Y, Yokoyama S, Muto A, Himeno H. 2009. Removal of a ribosome small subunit-dependent GTPase confers salt resistance on *Escherichia coli* cells. RNA 15:1766-74.

27. Lee EH, Kornberg A. 1991. Replication deficiencies in *priA* mutants of *Escherichia coli* lacking the primosomal replication n' protein. Proc Natl Acad Sci U S A 88:3029-32.

28. Nurse P, Zavitz KH, Marians KJ. 1991. Inactivation of the *Escherichia coli* *priA* DNA replication protein induces the SOS response. J Bacteriol 173:6686-93.

29. McCool JD, Ford CC, Sandler SJ. 2004. A *dnaT* mutant with phenotypes similar to those of a *priA2*::kan mutant in *Escherichia coli* K-12. Genetics 167:569-78.

30. Schmitt E, Tanrikulu IC, Yoo TH, Panvert M, Tirrell DA, Mechulam Y. 2009. Switching from an induced-fit to a lock-and-key mechanism in an aminoacyl-tRNA synthetase with modified specificity. J Mol Biol 394:843-51.

31. Kimura S, Hubbard TP, Davis BM, Waldor MK. 2016. The Nucleoid Binding Protein H-NS Biases Genome-Wide Transposon Insertion Landscapes. mBio 7:e01351-16.
