# Supplementary figures and images for: Revealing Causes for False-Positive and False-Negative Calling of Gene Essentiality in Escherichia coli Using Transposon Insertion Sequencing
Source: mSystems. 2022 Dec 12;8(1):e00896-22. doi: 10.1128/msystems.00896-22 (PMC9948719; doi:10.1128/msystems.00896-22)

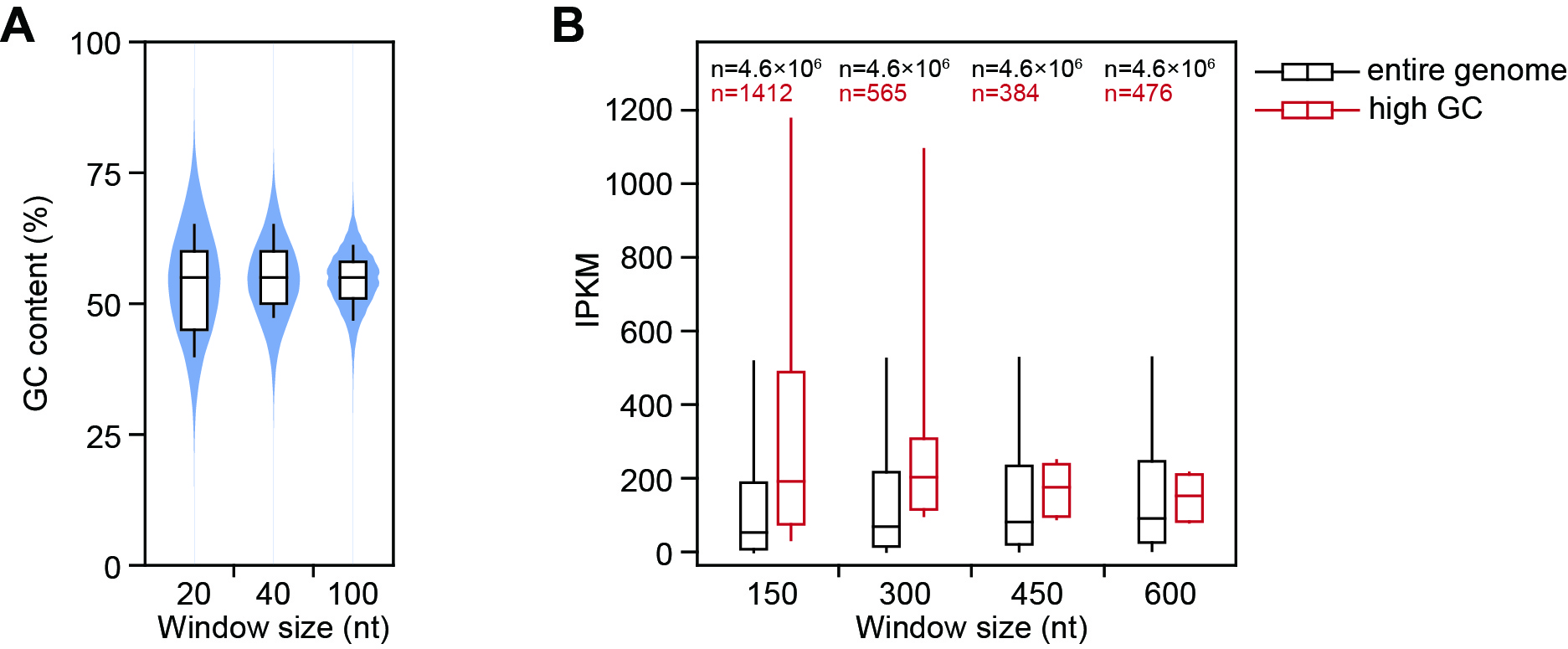

Supplement: FIG S1 [file msystems.00896-22-s0006.jpg]

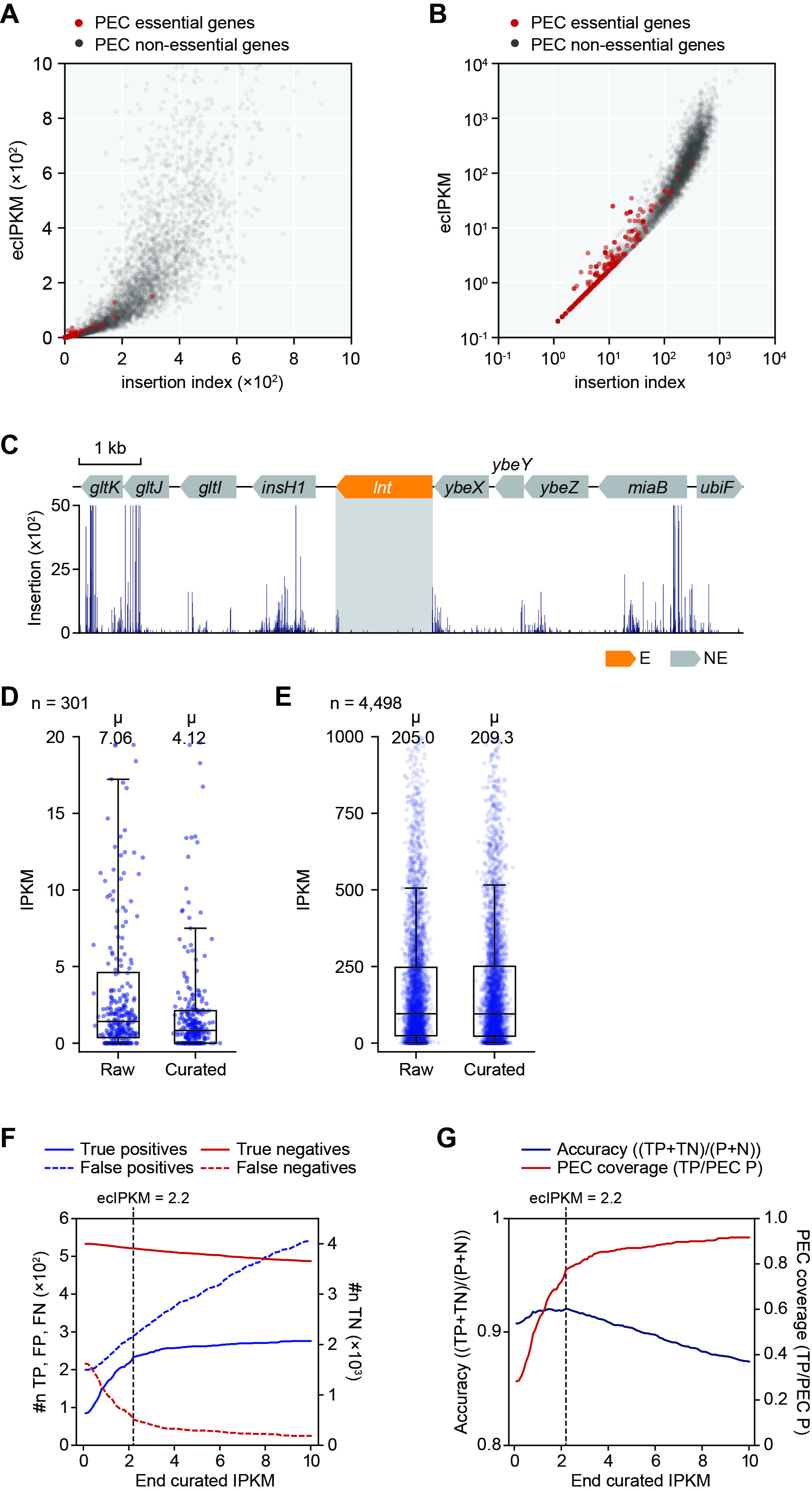

Supplement: FIG S2 [file msystems.00896-22-s0007.jpg]

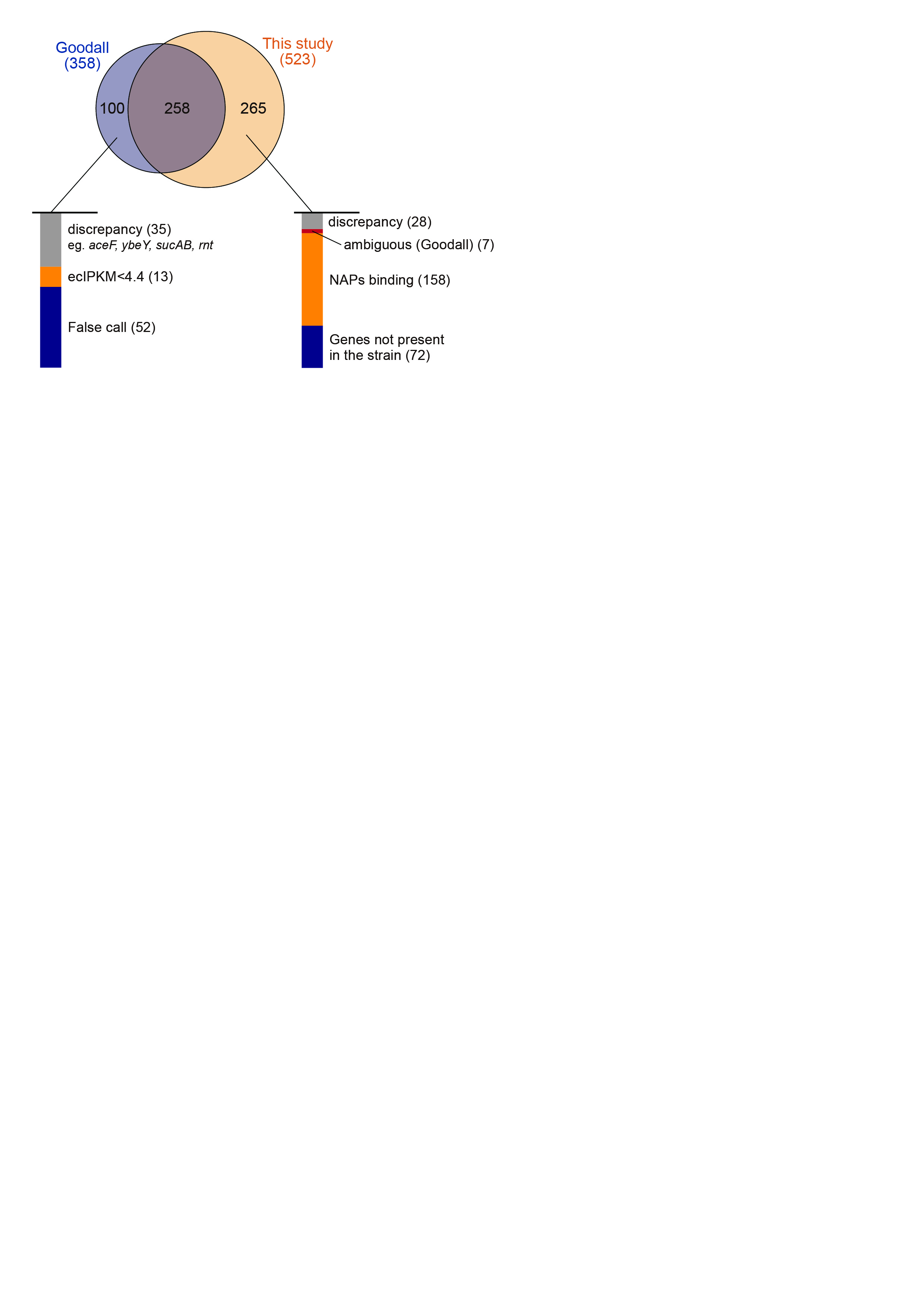

Supplement: FIG S3 [file msystems.00896-22-s0008.jpg]

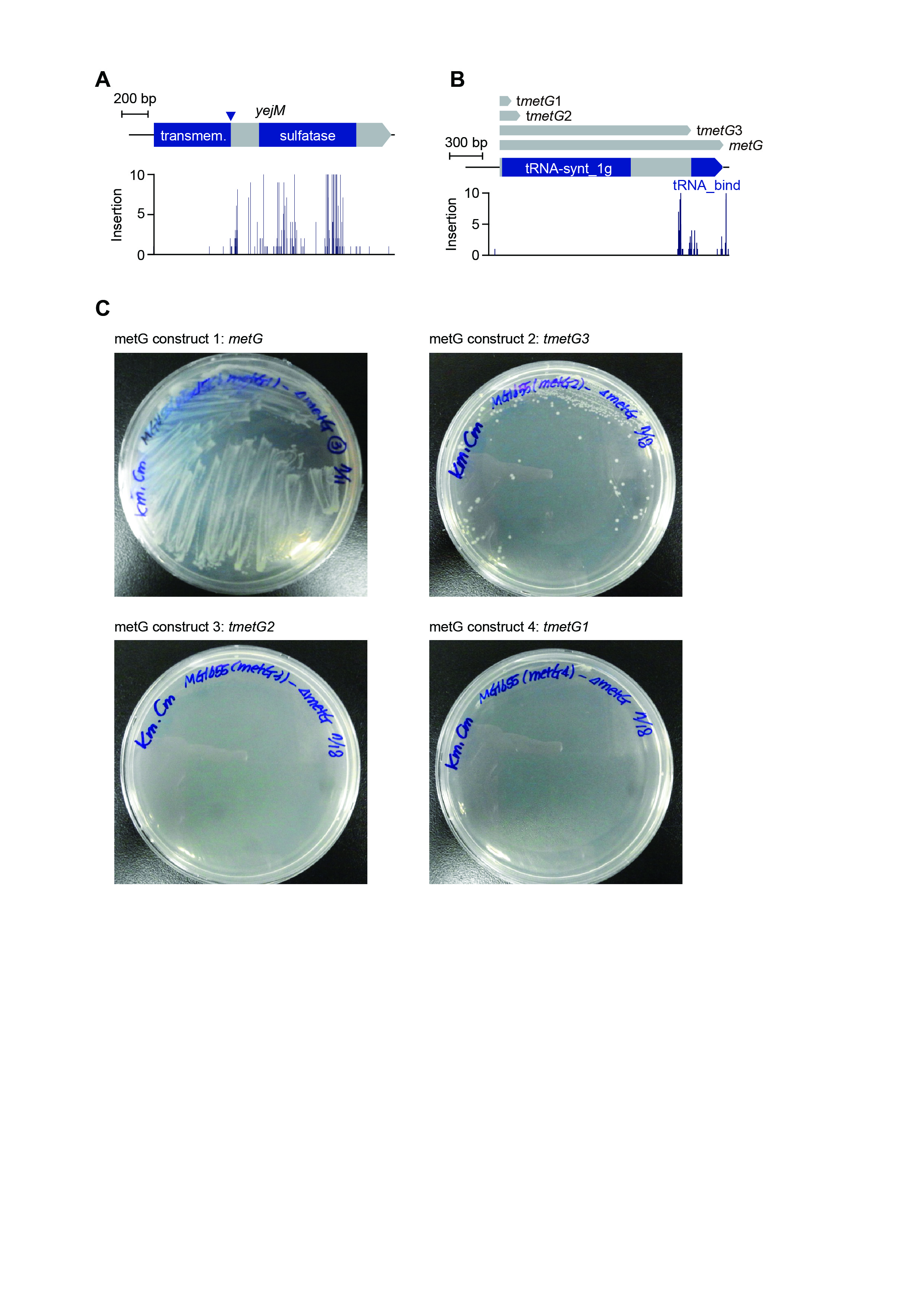

Supplement: FIG S4 [file msystems.00896-22-s0009.jpg]

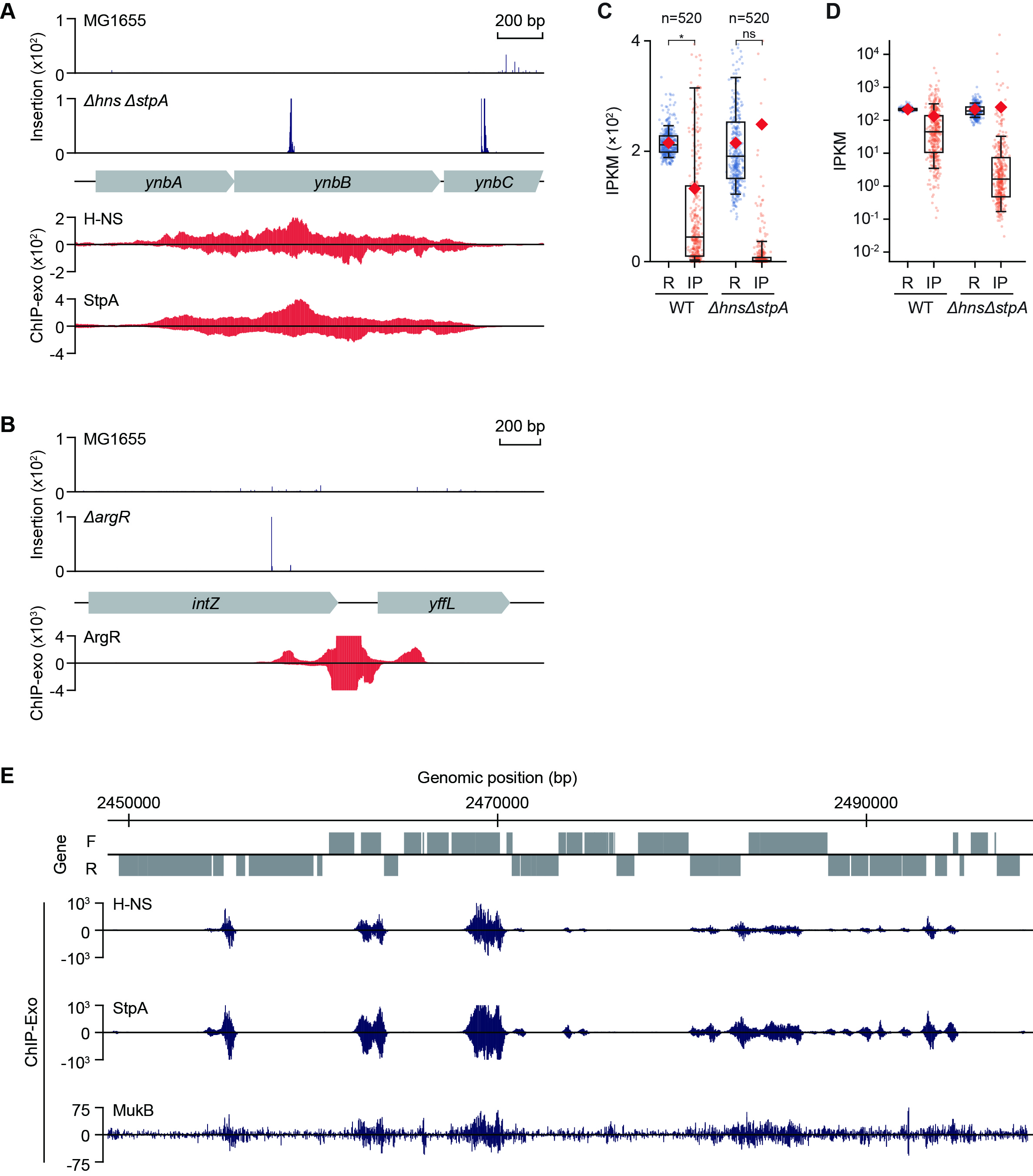

Supplement: FIG S5 [file msystems.00896-22-s0010.jpg]
